# Role of Omega-Hydroxy Ceramides in Epidermis: Biosynthesis, Barrier Integrity and Analyzing Method

**DOI:** 10.3390/ijms24055035

**Published:** 2023-03-06

**Authors:** Fei Ge, Keyan Sun, Zhenlin Hu, Xin Dong

**Affiliations:** School of Medicine, Shanghai University, Shanghai 200444, China

**Keywords:** omega-hydroxy ceramides (ω-OH-Cer), corneocyte lipid envelope (CLE), mass spectrometry (MS) analysis, integrity of skin barrier, skin care

## Abstract

Attached to the outer surface of the corneocyte lipid envelope (CLE), omega-hydroxy ceramides (ω-OH-Cer) link to involucrin and function as lipid components of the stratum corneum (SC). The integrity of the skin barrier is highly dependent on the lipid components of SC, especially on ω-OH-Cer. Synthetic ω-OH-Cer supplementation has been utilized in clinical practice for epidermal barrier injury and related surgeries. However, the mechanism discussion and analyzing methods are not keeping pace with its clinical application. Though mass spectrometry (MS) is the primary choice for biomolecular analysis, method modifications for ω-OH-Cer identification are lacking in progress. Therefore, finding conclusions on ω-OH-Cer biological function, as well as on its identification, means it is vital to remind further researchers of how the following work should be done. This review summarizes the important role of ω-OH-Cer in epidermal barrier functions and the forming mechanism of ω-OH-Cer. Recent identification methods for ω-OH-Cer are also discussed, which could provide new inspirations for study on both ω-OH-Cer and skin care development.

## 1. Introduction

In the history of organic evolution, creatures have covered themselves with skin to isolate their inner organs from the outer environment [1]. As one of the biggest organs in the human body, the skin has several essential functions. Most mechanical injury would be fatal if there is no skin coverage. Furthermore, survival on dry land requires a sustainable moisture containment system in the stratum corneum (SC), which can protect against the loss of body fluid and electrolytes [1,2]. The mammalian epidermis is a kind of multi-layered epithelium, which maintains its self-renewal ability under dynamic equilibrium and injury conditions by maintaining the mitotic active cell groups in hair follicles and the innermost basal layer. The SC functions as a barrier for the human body, and is formed naturally with a linear differentiation process. In this process, basal cells differentiate from a basal layer into spinous cells in the spinous layer, which in turn develop into enucleated granular cells in the granular layer, and subsequently function into squames in the SC [3]. The dysfunction of the SC will cause a disrupted skin barrier, exposing patients to water loss and skin inflammation, all of which would cause severe dermatological conditions such as atopic dermatitis, psoriasis vulgaris, and xeroderma pigmentosum [4].

During the skin-cornification process, lipids are transported to the extracellular space by lamellar bodies containing phospholipids, glycosylceramides, sphingomyelin, cholesterol, and enzymes [5,6]. Enzymes are known as metabolizers of lamellar lipids, and form the final lipid components of SC. Generally, human SC is composed of 50% ceramides (Cer), 25% cholesterol, and 15% free fatty acids (FA) [7]. As the most abundant component of SC, Cer has attracted the attention of researchers. In SC, the lipids derived from sebocytes, keratinocytes and microorganisms function as “mortar” for squames. The strong protective barrier of skin is built via lipids binding squames together [8]. Certain lipid components of SC will maintain a balance of inner homeostasis. To date, the cause of skin barrier disruption is poorly understood. In the meanwhile, the disruption of skin barrier integrity was confirmed as a mechanism for skin dermatoses [9]. Supplementation of Cer assists in the formation of the permeability barrier, which in turn can heal damage to the skin barrier [10]. Severe epidermal barrier perturbation occurs with selective ablation of ceramides in the epidermis [11]. Moreover, as a secondary lipid messenger, Cer and/or its metabolites mediate several cell signaling processes, including growth, differentiation, senescence, necrosis, proliferation, and apoptosis [12]. In current clinical treatment for skin barrier integrity, Cer has become a preferred alternative to corticosteroids, of which the latter might cause side-effects such as high blood pressure, headache, and in terms of skin, barrier weakening [13]. A recent review by Kono et al. [14] reveals 21 positive reports, indicating that external ceramide-containing preparations have effects on improving dry skin and barrier function. In these reports, formulations containing Cer have been shown to reduce transdermal water loss, improve stratum corneum structure, and/or increase stratum corneum fat content. These reports therefore detail the efficacy of ceramide-containing formulations.

**Figure 1:**
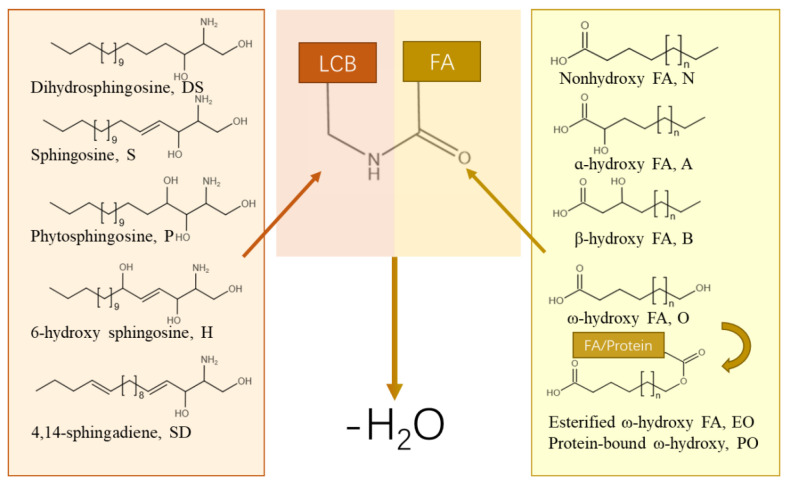
Structures and nomenclature for Cer classes in mammals.

The Cer molecule is composed of a long-chain base (LCB) and an FA attached by an amide bond [6]. Various combinations of LCB and FA make the molecular structure of Cer to differ from other molecules. For mammals, there are five types of LCB, including sphingosine (Sph), dihydrosphingosine (DS), phytosphingosine (PS), 6-hydroxy sphingosine (H), and 4,14-sphingadiene (SD), as well as four types of FA, including nonhydroxy FA (N), α-hydroxy FA (A), β-hydroxy FA (B), and ω-hydroxy FA (O) [15]. The structures and nomenclature for Cer classes in mammals are illustrated in Figure 1. Sph-based Cer will be the focus of the following discussion. In this review, the nomenclature of ceramides follows the report of Motta et al. [16] and Robson et al. [17]. The name of each Cer is a combination of a letter representing the type of FA, and another one representing the type of LCB. For example, Sph ceramides with α/β/ω-hydroxylated FA are designated as AS/BS/OS. Hydroxylation of LCB in ceramides introduces multiple variations in biomolecule functions. For instance, the absence of AS on the myelin sheath membrane leads to a loss of long-term stability of the myelin sheath, and eventually leads to demyelination [18,19]. The quantitative analysis of B in biological cycles evaluates the energy fatty acid oxidation and interrelated pathways. In other words, a regular and proper BS level indicates the body’s ability to deal with fasting states, as well as normal cell metabolism and maintenance of cell energy supply [20]. When it comes to OS, the most noticed function is its critical role in maintaining the integrity of SC. O-type FA is hydroxylated at the ω position of carbon chain, which is located at the end of carbon chain. Acylated with LCBs, O-type FA forms ω-hydroxyceramides (ω-OH-Cer), namely, OS/ODS/OPS/OH/OSD. This unique hydroxyl position gives ω-OH-Cer surfactant properties, and chances to combine with proteins or other FAs with the hydroxyl at the ω position of ω-OH-Cer.

In SC, keratinocytes are surrounded by a highly crosslinked protein network, also known as the cornified lipid envelope (CLE) [21]. ω-OH-Cer is the primary lipid component of CLE, which is covalently attached to the outer surface of the cornified envelope and connected with involucrin, forming lipids in SC [22]. ω-OH-Cers are synthesized in the SC (the outermost layer of the epidermis) from precursor molecules called glucosylceramides. The synthesis of omega-hydroxy ceramides is mediated by a family of enzymes called cytochrome P450 oxidases, which add hydroxyl groups to the ceramide molecules. These hydroxyl groups are typically added to the omega (ω) position of the fatty acid chain of the ceramide molecule, hence the name “ω-OH-Cers” [23]. The hydroxyl group at the ω position can be esterified with other molecules to form esterified FA, or be attached to a protein for the modification of protein-bound FA. Early in 1986, Philip et al. [24] reported that FA, ω-hydroxyacids, and ω-hydroxyacylSphs in mammals are covalently attached to macromolecules, where 60% of the hydrolysis products of these lipids comprises ω-hydroxyacylSph or OS. Acylceramide, formed by acylation of ω-OH-Cer, functions as a molecular rivet. It holds the extracellular bilayers, where the ω-hydroxyacyl chain spans one of the lipid bilayers and the linoleate tail is inserted into another bilayer [25]. The cutaneous permeability barrier is therefore constructed by matured corneocytes to form a moisture-containment system. The significant role of ω-OH-Cer in maintaining the epidermal barrier function was only discovered after years of clinical cases. Numerous abnormal barrier conditions, including malformation of the cornified lipid envelope [26], atopic dermatitis [22], and ultraviolet burns [22], are all due to disturbed ω-OH-Cer biosynthesis, which is huge threaten to skin barrier integrity. ω-OH-Cer contributes to this barrier function by forming lamellar structures that help to seal the spaces between corneocytes (the flattened, dead cells that make up the SC). These lamellar structures are formed by the interdigitation of long-chain ceramides, cholesterol, and free fatty acids, and they provide a water-repellent surface that helps to prevent water loss from the skin [27].

Therefore, quantitation and identification of omega-hydroxyceramides in the skin can provide important insights into the mechanisms underlying skin barrier function. In particular, the development of analytical methods to quantitate ω-OH-Cer has enabled researchers to assess the levels of ω-OH-Cer in healthy and diseased skin, providing valuable insights into the role of ω-OH-Cer in skin function and disease [28]. The analysis of omega-hydroxyceramides in the skin involves different steps, including separation and compound identification. Separation of ω-OH-Cer provides the potential of studying this Cer with an independent view. Compound identification with separated ω-OH-Cer provides a more specific identification, which can possibility be used for further quantitation [2,6,18]. These two steps, along with the analyzing methods, are valuable tools for understanding the biological functions of the ω-OH-Cer molecule, as well as for the study of the pathology and biology of ω-OH-Cer molecules [6].

In this review, the detailed molecule mechanism of SC recovery from acute barrier disruption is discussed, in order to evaluate the efficiency of ω-OH-Cer in CLE development. This review is organized as follows: (1) mechanism of epidermal barrier recovery; (2) the biosynthesis of ω-OH-Cer; (3) ω-OH-Cer functions as a molecular rivet in CLE development; (4) current identification studies of ω-OH-Cer and challenges; (5) conclusions.

## 2. Mechanism of Epidermal Barrier Recovery

**Figure 2:**
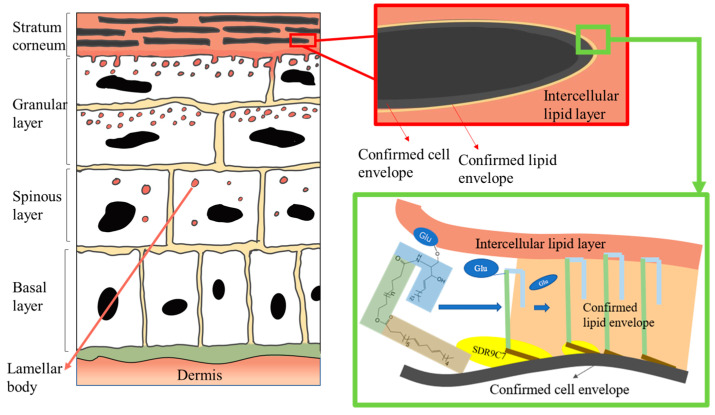
Schematic diagram of epidermal keratinocyte differentiation. Structure of the epidermal barrier and how the CLE is formed, in detail.

The mammalian epidermis maintains renewable ability under conditions of injury or homeostatic status, via population of mitotically active cells in hair follicles and the innermost basal layer [28,29]. The epidermal barrier is the outermost layer of the skin, and it plays a critical role in protecting the body from external insults and preventing water loss. When the epidermal barrier is damaged, the skin becomes more permeable and loses its ability to retain moisture. This can lead to skin dryness, inflammation, and other skin disorders [27].The forming process, as well as the recovering process, of the epidermal barrier are dynamic and similar. The detailed structure of the epidermal barrier is pictured in Figure 2. As the beginning sign of terminal differentiation, basal cells withdraw from cell cycles concomitantly and leave the basement membrane. Differentiation of basal cells to spinous cell leads to the next stage of epidermal keratinocytes, where the durable cytoskeleton frame of keratin filaments is reinforced, in order to gain sufficient mechanical strength for the potential physical impact. Following that, the spinous cells develop into granular cells. In granular layer, lamellar bodies produce lipids and herein assemble CLE, the highly crosslinked protein network, via sequential incorporation of precursor proteins underneath the plasma membrane [6]. In this process, epidermal keratinocytes go through most of their cell cycle and are close to the disintegration of their cell membranes [30]. The subsequent calcium influx activates the transglutaminase (TGM) enzyme to crosslink Cers with CLE proteins, forming a sac surrounding the keratin fibers, as shown in Figure 2. One of the protective elements of the epidermal barrier is derived from this tough, insoluble structure. As the dynamic process occurs from the inner layers to the surface, the final stage of the epidermal keratinocyte life cycle occurs in the SC, where lipids derived from sebocytes, keratinocytes, and microorganisms will function as the “mortar” for squames. 

For physical skin damage recovery, all those mentioned cells are included, and this dynamic process is similar to the process of epidermal barrier-forming, as mentioned before, but with certain differences, including inflammation. The process of wound healing occurs in five overlapping stages, namely hemostasis, inflammation, proliferation, re-epithelization, and fibrosis [31,32]. In inflammation stage, which follows initial hemostasis, the innate immune system helps to remove dead tissue and defend the body from invading pathogens [33]. The reconstruction of damaged tissue involves the proliferation and re-epithelization processes, which include collagen synthesis, extracellular matrix formation, and restoration of the vascular network [33,34]. Basal keratinocytes migrate continuously to the SC layer to rebuild physical barrier [31]. The final stage of wound healing is the formation of functionally and visually intact skin. These overlapping wound individual healing stages are complex and their processes are dependent on one another; disruption in any of the processes can induce a hypertrophic scar with long-lasting pruritis [32]. Hypertrophic scar formation brings a risk of keloids, which is benign hyperproliferation of fibroblasts [35]. Sung et al. demonstrated that growth of fibroblasts can be inhibited by Cer via apoptosis and supposedly Sph; the metabolic product of OS might have cytotoxic effects on growth of keloid fibroblasts [36]. Therefore, ω-OH-Cer not only plays an irreplaceable role in SC formation, but also is beneficial for epidermal barrier construction.

**Figure 3:**
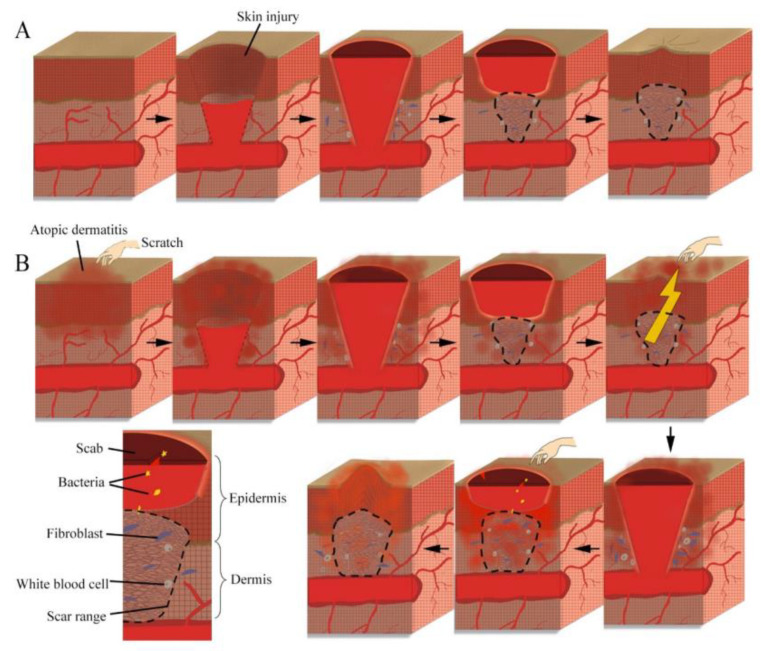
Schematic diagram of epidermal barrier recovery. (**A**) Epidermal barrier recovery due to physical skin damage. (**B**) Epidermal barrier recovery due to a lesion from atopic dermatitis.

Beside open skin injuries, recovery of the skin barrier from atopic dermatitis is another issue of great interest, which is shown in Figure 3. Atopic dermatitis is characterized by inflammation and chronic itching in skin. Barrier dysfunction induces an inflammatory environment and skin dryness, lowering the itch threshold [37]. Pruritus in atopic dermatitis generally causes scratching on the affected area. Scratching breaks down fragile skin barrier and triggers Type 2 inflammatory responses that could exacerbate itch sensitization. Due to continuous damage accrued in the itch–scratch cycle and pre-existing barrier dysfunction, the five overlapping stages discussed in the preceding paragraph will be interrupted, breaking the healing plan [38]. Fortunately, certain treatments can relieve these symptoms. Of numerous studies based on topical therapy, systemic agents, or biologics, the main goal for atopic dermatitis management is to maintain the integrity of the skin barrier [39,40,41]. Intact CLE not only prevents the loss of natural moisturizing, but also helps in forming a proper orientation of the intercellular lipid lamellar structure, by interdigitating with the intercellular lipids [42]. Attached to the outer surface of the cornified envelope, ω-OH-Cer, symbolized by OS in CLE, is linked to involucrin to function as lipid components of SC. ω-OH-Cer forms lipids in the SC in unique ways that other ceramides cannot replace. Patients suffering from a lack of ω-OH-Cer or its related enzymes are at an extremely high risk of atopic dermatitis, harlequin ichthyosis [43], psoriasis [44], and other diseases caused by skin barrier dysfunction [45,46]. Mutations in ω-OH-Cer synthesis or condensing-related gene have been shown to be causes of congenital ichthyoses and ichthyosis syndromes. These ichthyosis conditions can only be modified via ω-OH-Cer supplementation [47]. There has been no successful trial on non-ω-hydroxylated ceramides on modifying congenital ichthyoses or ichthyosis syndromes. Moreover, the specific mechanism for epidermal injury caused by UVB is the breakage of the lipid bond between ω-OH-Cer and other lipids in SC [23]. To assist in epidermal barrier recovery, synthetic ω-OH-Cer has been applied widely in order help regain the moisture containment system in animal experiments [48,49]. All in all, ω-OH-Cer supplementation is not an official treatment for any specific disease, but the existence of ω-OH-Cer builds a strong barrier for the SC.

## 3. The Biosynthesis of ω-OH-Cer in Epidermal Barrier

**Figure 4:**
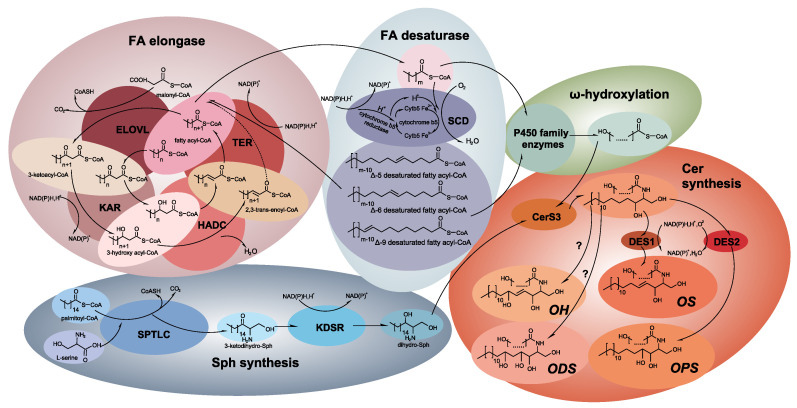
Overall methodology for de novo ω-OH-Cer biosynthesis. Pathways involved in de novo ω-OH-Cer biosynthesis, the detailed product molecule structure, and the key enzymes are shown.

ω-OH-Cer molecules were synthesized via de novo synthesis and salvage pathways. To detail the whole process of ω-OH-Cer synthesis, the FA and Cer synthesis processes are described individually below. Figure 4 is a vivid emerge of this whole process.

### 3.1. The Synthesis of Long-Chain FA (LCFA) for ω-Hydroxylation

The synthesis of FA is an iterative process, where FA chain length is strictly under control. Unlike proteins and nucleic acids, the monomeric unit of FA has less freedom and the chain length is more complex to predict, since this process is defined via FA synthase (FAS) instead of mRNA or DNA templates. During FA synthesis, there is no specific boundary for FA chain elongation, hydroxylation, and desaturation. Therefore, the variation of the FA chain is diverse. Despite the numerous FA products garnered using FA biosynthesis, the natural ω-hydroxylation on FA only occurs on FA chains with more than 16 carbon atoms [24].

Upon the onset of long-chain FA (LCFA) synthesis, FASs commonly release palmitic acid (C16:0) and stearic acid (C18:0), or the coenzyme A (CoA) derivatives thereof [50]. Most elongations of FA are achieved based on those products. FA elongations circulate via four processes: condensation, reduction, dehydration, and reduction [51]. In the first step, enzymes embedded in the endoplasmic reticulum elongate FAs, in order to convert FAs into acyl-CoAs. FA elongase catalyzes the production of 3-ketoacyl-CoA by condensing acyl-CoA with malonyl-CoA. Mammals have seven FA elongases with characteristic substrate specificity, named after the elongase of very-long chain fatty acid 1–7 (ELOVL1–7) with specific substrates [50]. In the second step, 3-ketoacyl-CoA is reduced to 3-hydroxy acyl-CoA by 3-ketoacyl-CoA reductase, named KAR in mammals [52]. Later, 3-hydroxyacyl-CoA dehydratase (HACD1-4) dehydrates 3-hydroxy acyl-CoA into 2,3-trans-enoyl-CoA, in order to take the LCFA synthesis to the last step. Then, 2,3-trans-enoyl-CoA reductase (TER) takes over in order to catalyze the formation of elongated acyl-CoA.

Besides saturated fatty acid, the synthesis of unsaturated FA is also interesting. Firstly, the unsaturation process of elongated acyl-CoA or FAs should be clarified. CoA desaturases introduce a double bound to a specific location on the acyl-CoA chain, where the biological properties are complexed for further biofunctions. CoA desaturase is classified in accordance with the location on which the double bound is introduced (Δ number to indicate the location on carbon chains). In mammals, activities of Δ9, Δ6, and Δ5 CoA desaturases are observed [52]. All the desaturases found in mammals are only stearoyl-coA desaturases (SCDs). The other desaturase family, the so-called fatty acid desaturase (FADS) family, is not present in mammals. SCDs are endoplasmic reticulum enzymes, which catalyze the saturated FAs, synthesized de novo or from diary intake, into monounsaturated FAs. The desaturating process is essentially the transmission of hydrogen ions (H^+^). The combination of nicotinamide adenine dinucleotide (NADH), flavoprotein cytochrome b5 reductase, and the electron acceptor cytochrome b5 provides one molecular oxygen of adequate hydrogen ions to form two molecules of H_2_O, half from the transmission of hydrogen ions and half from substrates [53]. The preferred substrates of SCD are palmitoyl (C16:0)- and stearoyl (C18:0)-CoA, which are desaturated into palmitoleoyl (C16:1)- and oleoyl (C18:1)-CoA [54].

Noticeably, elongase activities and the process of FA occur in parallel until the chain length hits C26, when ELOVL4 takes over the elongase process and the desaturase process barely appears. The longer the carbon chain grows, the weaker the control SCD has on them. Therefore, though FAs can be catalyzed five or six times, the supplementation of essential FAs, such as docosahexaenoic acid (DHA, C22:6), in the diet is important [55].

The ω-hydroxylation of FA is not limited to a certain chain length. Although it is not thoroughly clarified, one of the credible theories based on the activity of microsomal cytochrome P450 enzymes is put into practice in multiple studies [50,56]. Cytochrome P450 family, a heme-containing monooxygenase, has a special ability to catalyze the hydroxylation of the terminal carbon atoms in the inactivated alkyl chain [57]. About 300,000 sequences of cytochrome P450 have been identified since it was first discovered in the early 1960s [58]. ω-hydroxylation of FA includes two main P450-dependent mechanisms. FAs with carbon chain longer than C30 are produced from a chain extension of palmitic acid (C16:0) and then hydroxylated by the P450 enzyme. In contrast, FAs with shorter carbon chains are directly ω-hydroxylated by the P450 family [24]. In 2000, Behne et al. [48] adapted amino benzotriazole, a chemical relative with notable P450 inhibitory activity, into cultured human keratinocytes, and found that it demonstrated significant decline in ω-hydroxylated FA and led to increased water loss in the epidermal barrier. This work proved the vital role P450 plays in ω-hydroxylated FA formation, as well as in epidermal health. Six years later [59], mutations on a new gene FLJ39501, which encodes CYP4F22 (cytochrome P450, family 4, subfamily F, polypeptide 22), were found in 21 patients with autosomal recessive congenital ichthyosis, in four countries. Ohno et al. [60] further delved into the investigation of FLJ39501, the FA ω-hydroxylation gene, to prove that CYP4F22 is a bona fide ULCFA ω-hydroxylase required for acyl Cer production to enhance skin permeability barrier function. In 2019, a more detailed theory of lamellar ichthyosis based on a missense mutation in exon 8, CYP4F22 Arg243Leu, was predicted to be a functionally defective variant via in silico analysis [61]. An abnormality of cytochrome P450-related genes in human mutations can further induce obvious epidermal damage, which will expose hosts to hyperkeratosis, mild acanthosis, or parakeratosis symptoms [24,48,56,57,58]. Not only meaningful for ω-hydroxy FA biosynthesis, fungal P450 and its outstanding ω-hydroxylation performance shed light on the artificial synthesis of ω-hydroxy FA in commercialized synthesis [50]. Despite the related knowledge being still limited, further studies on FA ω-hydroxylase are still carrying on.

### 3.2. The Synthesis of ω-OH-Cer in the Epidermal Barrier

The onset of LCB biosynthesis in ω-OH-Cer is the condensation of palmitoyl (C16:0)-CoA and L-serine by serine palmitoyltransferase (SPTLC), the product of which is 3-ketodihydro-Sph. In the following, 3-ketodihydro-Sph reductase (KDSR) reduces 3-ketodihydro-Sph to dihydro-Sph. Dihydro-CER D4-desaturase (DES1) desaturates the dihydro-Sph between C4 and C5 to form a double bound, from which a Sph base is completed [62]. Dihydro-Sph can also be hydroxylated at the C3 position by dihydro-CER hydroxylase (DES2) to complete a PS base, which is another LCB, to synthesize ω-OH-Cer [62].

The synthesis of ω-OH-Cer follows the regular synthesis process of Cer synthesis, where Cer synthase (CerS) participates in the main esterifying process. Specifically, CerSs have preference for certain fatty acyl-CoAs. For example, CerS5 and CerS6 are active with C14:0-C18:0 fatty acyl-CoAs [63,64,65]; CerS1 is active with C16:0-C18:0 fatty acyl-CoAs [64]; CerS4, CerS2, and CerS3 are active with C18-C24 fatty acyl-CoAs [64,65,66]. To produce ceramides with longer chain lengths, CerS2 and CerS3 can keep active for fatty acyl-CoAs up to C26 [66,67]. The lack of CerS3 in mice directly induces the complete loss of ultra-long-chain ceramides (>C26) and ω-OH-Cer [67]. In this aspect, the ω-OH-Cer de novo synthesis process in mammals is highly dependent on CerS3.

Other than being a de novo pathway, Cers can also be re-acylated by the salvage pathway via CerSs [56].

## 4. ω-OH-Cer Functions as Molecular Rivet in CLE Development

As mentioned before, SC mainly consists of terminally differentiated keratinocytes, which are embedded into the extracellular lipid matrix. During terminal differentiation, the cornified envelope (CE), a crosslinked protein structure, replaces the plasma membrane [30]. CLE is a monolayer of lipids bound covalently to CE. Being the interface of hydrophilic corneocytes and the lipophilic extracellular lipids, CLE is vital for skin barrier stability [26]. The surfactant properties of CLE are mainly based on its complex lipid components, of which ceramide accounts for the majority. ω-OH-Cer stands out for its hydroxylated group at the end of its carbon tail, with which CE proteins are connected by ester linkage [48]. 

The CLE is the product of keratinocytes during the differentiation process from specific lipid vesicles, which comprises lamellar bodies in the upper part of viable epidermis keratinocytes [2,6,68]. Within the latter differentiation process, Cer precursors (glycosylated or phosphocholinated ceramides), together with their converting enzymes, are released to extracellular space. Concurrently, the CLE forms via enzymes involved in binding Cer with CE, co-located at SC [68,69].

The binding of Cer with CE is based on the covalent binding of ultra-long-chain (ULC) Cer (ULC-Cer) to proteins on the surface of CE, as shown in Figure 2. ULC-Cer is derived from ultra-long-chain acylceramide, a Cer species in which the N-acyl chain is composed of ω-hydroxylated ULC-fatty acids esterified with linoleic acid (C18:2) to form esterified ω-hydroxy sphingosine (EOS), esterified ω-hydroxy phytosphingosine (EOP), and esterified ω-hydroxy 6-hydroxy sphingosine (EOH) [70]. The formation of CerEOS and CLE, as well as the genes/molecules involved, have been researched by studies on water-loss-related diseases, such as ichthyosis [69]. However, the mechanism of CerEOS binding to proteins on the outer surface of CE is still not well understood.

In 2020, Takerchi hypothesized that the loss of SDR9C7, which is generally responsible for ichthyosis, is the key for CerEOS binding to proteins [71]. It was conjected that after 12R-LOX catalyzes CerEOS to form 9R-hydroperoxy-CerEOS, eLOX3 takes over to further catalyze 9R-hydroperoxy-CerEOS into epoxy-alcohol-CerEOS. After these processes, SDR9C7 oxidization happens, resulti into epoxy-enone-CerEOS. The non-enzymatic binding to proteins on the extracellular surface of CE relies on this epoxy-enone [72]. Finally, the covalently bound epoxy-enone-CerEOS forms CerEOS-bound protein by an unknown mechanism (probably via the Michael addition reaction or Schiff base and pyrrole formation) [71]. In 2021, Youssefian et al. [73] conducted a clinical and molecular characterization work of 19 patients with autosomal recessive congenital ichthyosis, in five families with SDR9C7 gene mutation. The apparent knockdown of SDR9C7 coupled with ichthyosis symptoms indicates a strong link between SDR9C7 mutations and ichthyosis. Downregulation of SDR9C7 by small interfering RNA techniques in three-dimensional organotypic skin construction in in vitro keratinocytes also led to similar morphological and histological abnormalities in ichthyosis patients [73]. Takeichi et al. [72] carried out liquid chromatography–mass spectrometry (LC-MS) quantitative assays on epoxy-enone-CerEOS in SDR9C7-mutated patients and SDR9C7-KO mice. The disappearance of epoxy-enone-CerEOS, compared with a higher abundance of other acylceramides related to the lipoxygenase pathway in these cases, verified ADR9C7 as being a critical requirement for production of epoxy-enone-CerEOS, which is known for its nonenzymatic coupling to proteins [72]. Mutations of the SDR9C7 family are strongly associated with Mendelian disorders of cornification, a highly heterogeneous group of diseases [74]. Collectively, constructions of CerEOS with proteins, which ensure epidermal barrier integrity, are severely dependent on SDR9C7.

## 5. Current Identification Studies of ω-OH-Cer and Challenges

In 1986, Philip et al. [24] reported the irreplaceable position of hydroxylated ceramides in CLE lipids. In the same year, Philip et al. continued to verify that the bound lipids are mostly composed of ω-OH-Cer (53.3%) and ω-hydroxyacid-containing (24.8%) Cer in human SC, via traditional quantitative thin-layer chromatography coupled with gas–liquid chromatography [75]. This innovative discovery, at the time, attracted much interest regarding ω-OH-Cer in the SC, and, therefore, gave a primitive guide for identification studies of ω-OH-Cer.

The identification and quantification of small biomolecules such as ceramides can be analyzed via a wide array of analytical methods, such as gas chromatography–mass spectrometry (GC-MS), LC-MS, and high-performance thin layer chromatography (TLC) coupled with MS detection. Herein, the following part will discuss these methods for ω-OH-Cer identification. Hopefully this will be an inspiration for further study. Conventional techniques, for instance, ultraviolet spectroscopy (UV) analysis, have been used as the standard method for ω-OH-Cer identification. However, the accuracy and stability of MS has gradually led to it becoming the technique of choice for structural analysis. Especially when it comes to isomer discrimination, the regular fragmentation makes the identification easier and more specific for detail picturing [76].

There is a small body of work reporting GC-MS for the qualitative study of ω-OH-Cer. Without derivation, lipids are fragile, since they are non-volatile and instable [77]. Commonly, the GC-MS analysis method for ω-OH-Cer identification is developed together with other ceramides. Since ceramides do not show sufficient volatility, derivation is essential for GC analysis. Over the years, methods have been used in order to realize perfect derivations, such as yielding trimethylsilyl derivatives [78] and permethylated ceramides [77]. Due to its complexity and instability, GC-MS commonly displays lower efficiency and adaptability for Cer analysis than LC-MS. In the early stage of Cer analysis, GC was chosen for analysis, due to its cheap cost [79,80]. Modifications of LC systems have drawn more researchers to lean towards LC-MS as the more preferred method for ω-OH-Cer analysis.

With the rapid development of lipidomics in recent decades, the number of LC-MS studies on ω-OH-Cer has gone through an exponential growth [81]. The LC separation system is based on different affinities of each component in two phases. The LC systems used can be divided into liquid–solid chromatography, liquid–liquid chromatography, and bonded-phase chromatography, according to the difference in stationary phases. The mostly adapted LC system comprises liquid–solid chromatography, where silica gel is applied as a filler in the columns, and bonded-phase chromatography, where micro silica gel is applied as a matrix for the columns [81]. One of the most important aspects of LC method optimization is to choose a suitable LC column. The choices of mobile phase and its matching ratio, column temperature, and flow rate are all variances for LC method optimization. Commonly, normal-phase (NP) LC and reverse-phase (RP) LC are all used to analyze ceramide. NP-LC distinguishes ceramides according to their hydrophilic functionalities [79], but it is more suitable for separating ceramides into their representative classes than coupling electrospray ionization (ESI), and achieves high sensitivity in mass spectrometry analysis. Furthermore, to separate ω-OH-Cers, a Cer class, NP-LC narrows the elution time to a narrow range, making it difficult to identify each molecule [82]. Conversely, RP-LC realizes the separation of ceramides based on their hydrophobic properties. Depending on mainly the carbon chain length and number of unsaturated bonds, RP-LC significantly raises the separation efficiency for weakly intrinsic hydrophilic biomolecules such as ω-OH-Cers. Likewise, RP-LC improves the higher peak capacity for lipid species [83].

TLC, a traditional and practical means for separation and quantification, has also been applied in ω-OH-Cer analysis for years [81,83]. By comparing the migration front (Rf) with known and available standards, the identification of the Cer class can be achieved [76]. However, TLC suffers as a technique due to the lack of maturity, and thorough and commercialized standards. Luckily, TLC coupled with further identification or validation, namely, MS, technology can overcome this problem.

MS is a method to analyze and identify samples by measuring and analyzing the mass-to-charge ratio (*m*/*z*) of sample ions. There are three main parts of MS: the ion source, mass analyzer and detector. The mass analyzer is the core component of the mass spectrometer, which determines the sensitivity, resolution, and accuracy. Prior to analysis, the sample must be ionized so that the sample molecules will be charged [80]. Then, the charged ions are first driven by an accelerated electric field and fly into the analysis electric field or magnetic field. Due to the difference in the quality of the sample itself and the ionization charge, the motion trajectories of the sample ions in the analytical electric field are also different, so different ions can be distinguished by analyzing the motion trajectories of the ions, and the information, purity and other characteristics of the sample can be qualitatively and quantitatively determined. The two ion modes in MS, which are the positive mode and negative mode, are separately adapted for their own utilities [84]. Commonly, ω-OH-Cers are analyzed under positive mode, due to its high sensitivity and ability to help characterize the protonated (i.e., [M+H]^+^) and lithiated (i.e., [M+Li]^+^) states of the molecular species at low collision energy range [81]. Negative mode stands out for its high efficiency characterizing molecular structures [85]. With the participance of acetic acid (or formic acid), ions in negative mode are detected as adduct [M+CH_3_CO_2_]^−^ ions, which are dissociated into [M−H]^−^ for further structure analysis.

Unfortunately, the diversification of analytical methods still has not garnered the best method for ω-OH-Cer detection. With more reports on ω-OH-Cer, this research gap will hopefully be fulfilled by further refined methods for the identification and separation of ω-OH-Cer.

## 6. Conclusions

ω-OH-Cer has gained increasing attention. The unique function of ω-OH-Cer makes this class of Cer molecules vital for epidermal barrier formation and reconstruction. With more experimental and clinical practices on the effect ω-OH-Cer has for skin integrity, the lipid research has now progress into a new stage, where detailed and specific sphingolipid classes should be further studied. In this review, the detailed mechanism of ω-OH-Cer biosynthesis and its role are discussed. Hydroxylation gives ω-OH-Cer more biological potential. Correspondingly, the identification of ω-OH-Cer in studies in recent years has gained more attention. MS technology for qualification in biological samples is impressive, but still faces many challenges. This review aimed to garner inspiration for more specific research on ω-OH-Cer in epidermal integrity.

## Data Availability

Data sharing not applicable.

## Abbreviations

A	α-hydroxy fatty acid	LCB	long-chain base
B	β-hydroxy fatty acid	LCFA	long-chain fatty acid
CE	cornified envelope	LC-MS	liquid chromatography-mass spectrometry
Cer	ceramide	MS	mass spectrometry
CerS	ceramide synthase	N	nonhydroxy fatty acid
CLE	corneocyte lipid envelope	NADH	nicotinamide adenine dinucleotide
CoA	coenzyme A	NP	normal-phase
DES	dihydro-CER D4-desaturase	O	ω-hydroxy fatty acid
DHA	docosahexaenoic acid	PS	phytosphingosine
DS	dihydrosphingosine	RP	reverse-phase
ELOVL	elongase of very long chain fatty acid	SC	stratum corneum
EOH	esterified ω-hydroxy 6-hydroxy sphingosine	SCD	stearoyl-CoA desaturases
EOP	esterified ω-hydroxy phytosphingosine	SD	4,14-sphingadiene
EOS	esterified ω-hydroxy sphingosine	Sph	sphingosine
ESI	electrospray ionization	SPTLC	serine palmitoyltransferase
FA	fatty acid	TER	2,3-trans-enoyl-CoA reductase
FAD	fatty acid desaturase	TGM	transglutaminase
FAS	fatty acid synthase	TLC	thin layer chromatography
GC-MS	gas chromatography-mass spectrometry	ULC	ultra-long-chain
H	6-hydroxy sphingosine	UV	ultraviolet spectroscopy
HACD	3-hydroxyacyl-CoA dehydratase	ω-OH-Cer	omega-hydroxy ceramides
KDSR	3-ketodihydro-Sph reductase		
